# Mosla Chinensis Extract Enhances Growth Performance, Antioxidant Capacity, and Intestinal Health in Broilers by Modulating Gut Microbiota

**DOI:** 10.3390/microorganisms12122647

**Published:** 2024-12-20

**Authors:** Wei Wang, Yuyu Wang, Peng Huang, Junjuan Zhou, Guifeng Tan, Jianguo Zeng, Wei Liu

**Affiliations:** 1 College of Animal Science and Technology, Hunan Agricultural University, Changsha 410128, China; wangwei_2023@stu.hunau.edu.cn (W.W.);; 2 Hunan Key Laboratory of Traditional Chinese Veterinary Medicine, Hunan Agricultural University, Changsha 410128, China; 3 College of Veterinary, Hunan Agricultural University, Changsha 410128, China; wangyuyu@yzwlab.cn

**Keywords:** mosla chinensis, broiler, antioxidant, gut microbiome

## Abstract

This study aimed to evaluate the effects of Mosla chinensis extract (MCE) on broiler intestinal health. A total of 240 1-day-old Arbor Acres (AA) broilers (balanced for sex) were randomly allocated into four treatment groups, each with six replicates of 10 chickens. The study comprised a starter phase (days 1–21) and a grower phase (days 22–42). The control group (C) received a basal diet, while the experimental groups were supplemented with low (S1, 500 mg/kg), medium (S2, 1000 mg/kg), and high doses (S3, 2000 mg/kg) of MCE. The results showed that MCE supplementation significantly improved average daily gain in broilers (*p* < 0.05) and reduced the feed-to-gain ratio in broilers. Additionally, MCE enhanced the anti-inflammatory and antioxidant capacity of broilers. In the duodenum and cecum, MCE significantly upregulated the expression of tight junction proteins Claudin-1, and Occludin, with the high-dose group showing the strongest effect on intestinal barrier protection (*p* < 0.05). There was no significant difference in ZO-1 in dudenum (*p* > 0.05). Microbial analysis indicated that MCE supplementation significantly reduced the Chao and Sobs indices in both the small and large intestines (*p* < 0.05). At the same time, the Coverage index of the small intestine increased, with the high-dose group demonstrating the most pronounced effect. Beta diversity analysis revealed that MCE had a significant modulatory effect on the microbial composition in the large intestine (*p* < 0.05), with a comparatively smaller impact on the small intestine. Furthermore, MCE supplementation significantly increased the relative abundance of Ruminococcaceae and Alistipes in the large intestine, along with beneficial genera that promote short-chain fatty acid (SCFA) production, thus optimizing the gut microecological environment. Correlation analysis of SCFAs further confirmed a significant association between the enriched microbiota and the production of acetate, propionate, and butyrate (*p* < 0.05). In conclusion, dietary supplementation with MCE promotes healthy growth and feed intake in broilers and exhibits anti-inflammatory and antioxidant effects. By optimizing gut microbiota composition, enhancing intestinal barrier function, and promoting SCFA production, MCE effectively maintains gut microecological balance, supporting broiler intestinal health.

## 1. Introduction

Antibiotic Growth Promoters (AGPs) refer to the practice of adding low doses of antibiotics to animal feed to enhance growth and improve feed efficiency. However, the prolonged use of AGPs has accelerated the development of antimicrobial resistance, posing significant health risks to both humans and animals [1]. In recent years, the overuse of antibiotics has led to increasing levels of resistance, prompting many countries to prohibit AGPs [2]. In 2020, the Ministry of Agriculture and Rural Affairs of China issued Notice No. 194, which mandates a comprehensive ban on the addition of antibiotics in animal feed, effective from 1 July 2020. In the United States, the use of AGPs has also garnered widespread attention, particularly in poultry and swine production. The U.S. Department of Agriculture (USDA) has implemented a series of policies aimed at encouraging farmers to reduce their reliance on AGPs. Alternative solutions, such as plant extracts, probiotics, and precision nutrition management, are gradually becoming mainstream [3]. Brazil, as an agricultural powerhouse, also faces the challenge of excessive AGP use. The Brazilian government has started taking measures to reduce the use of AGPs, especially in meat production [4]. Meanwhile, the European Union took the lead in 2006 by implementing a comprehensive ban on the use of AGPs, becoming the first region in the world to enforce such a ban. Since then, the EU’s livestock sector has gradually shifted to using probiotics, organic acids, yeast, and precise nutrition management as alternatives to maintain animal growth performance and health [5]. Consequently, there is an urgent need to identify green, safe, and healthy alternatives to antibiotics for livestock and poultry production. In modern livestock production, animals are frequently exposed to various external stressors such as temperature fluctuations, transportation, nutritional imbalances, and pathogenic infections. These stressors can trigger oxidative stress within the body, disrupting the balance between pro-oxidants and antioxidants, which ultimately weakens immune function [6]. Oxidative stress occurs due to an imbalance between pro-oxidants (such as hydrogen peroxide and superoxide) and antioxidants (such as glutathione, superoxide dismutase, etc.). Hydrogen peroxide (H_2_O_2_) is a typical pro-oxidant that exacerbates oxidative damage by inducing lipid peroxidation, protein oxidation, and DNA damage, thus impairing cell functions [7]. Antioxidants like glutathione neutralize free radicals through their thiol group, effectively reducing cellular damage caused by oxidative stress [8].Oxidative stress not only affects overall animal health but also impairs intestinal barrier function by reducing nutrient absorption and subsequently decreasing production performance [9]. The gut, as a key immune organ, serves as the primary site for nutrient absorption as well as the first line of defense against external pathogens [10]. Maintaining a stable gut microbiota is crucial for growth performance, immune function, and antioxidant capacity in animals [10].

As concerns over animal health and food safety grow, plant extracts have garnered significant attention as promising green feed additives. Rich in bioactive compounds such as essential oils, flavonoids, and phenolic compounds, these extracts exhibit potent antioxidant, antimicrobial, and immunomodulatory properties. For instance, *Curcuma longa* (turmeric) extract, which contains the phenolic compound curcumin, has been shown to possess strong antioxidant and anti-inflammatory effects, enhancing both immune function and overall health in livestock [11]. Likewise, Cinnamomum verum (cinnamon) bark extract has demonstrated efficacy against antibiotic-resistant bacteria and boosts immune responses through its anti-inflammatory actions [12]. Compared to synthetic chemicals and antibiotics, plant extracts are not only safe and environmentally friendly but also effectively mitigate the problems associated with antibiotic resistance [13]. Moreover, plant extracts can enhance overall animal health, thereby supporting the quality and safety of animal-derived food products [14]. *Mosla chinensis Maxim*, a member of the Lamiaceae family, is an erect herbaceous plant widely distributed in regions such as Jiangxi and Hunan in China, with notable medicinal value. Its primary bioactive constituents include essential oils and flavonoids [15], which possess antioxidant, antimicrobial, and insecticidal properties [16,17,18]. Given these characteristics, extracts from *Mosla chinensis Maxim* show great potential as feed additives for livestock and poultry, particularly in enhancing animal health and antioxidant capacity. However, research on its application in livestock production remains limited, especially in areas related to regulating antioxidant and immune factors, as well as improving gut microbiota in a systematic manner.

Given the promising properties of Mosla chinensis Maxim, this study hypothesizes that extracts of M. chinensis could provide beneficial effects on growth performance, antioxidant capacity, immune function, and gut microbiota in broilers. Specifically, it is anticipated that bioactive compounds in MCE, such as flavonoids and essential oils, may help alleviate oxidative stress, improve gut health, and serve as a green alternative to antibiotics.

In this experiment, 240 healthy, 1-day-old Arbor Acres (AA) broilers of uniform weight were selected to examine the effects of adding 500, 1000, and 2000 mg/kg of M. chinensis extract (MCE) to their diets. The study aims to evaluate its impact on growth performance, serum biochemistry, antioxidant capacity, immune function, and gut microbiota of broilers. This research will provide a novel perspective on utilizing plant extracts, such as M. chinensis extract, as green feed additives in poultry production, aiming to support gut health in poultry and promote sustainable antibiotic-free farming practices.

## 2. Materials and Methods

### 2.1. Extracts and Animals

*M. chinensis* was purchased from Anguo Herbal Medicine Market, and its extract was prepared in the Key Laboratory of Veterinary Traditional Chinese Medicine at Hunan Agricultural University. The primary active components of the extract were 23.08% total flavonoids and 0.57% rosmarinic acid. Diets for the experiment were prepared at the New Starting Point Feed Factory of the Hunan Changsha Institute of Animal Husbandry and Veterinary Medicine. We selected 240 one-day-old Arbor Acres (AA) white-feathered broilers with no significant difference in body weight, half male and half female, purchased from Hunan Shuncheng Industrial Co., Ltd (Ningxiang, China). Prior to chick arrival, the chicken houses were disinfected through formaldehyde-potassium permanganate fumigation. The broilers were housed in a three-tier caging system, with each cage containing 10 chickens, totaling 24 cages. Six cages constituted one replicate, and natural ventilation was provided with 24-hour access to clean drinking water. Relative humidity was maintained between 55% and 65%. Powdered feed was supplied ad libitum throughout the trial. The chicken houses were cleaned daily and regularly disinfected, and the chickens were vaccinated following a standard immunization schedule. All procedures were approved by the Animal Care and Use Committee of Hunan Agricultural University.

### 2.2. Disinfection Schedule and Immunizations

Before we moved the chicken cages out of the chicken house, we scrubbed them with 3% sodium hydroxide, cleaned them with a high-pressure water gun, and let them air-dry for 1 week. Then, we removed the plastic film from the chicken house, soaked it in a cleaning solution made of iodine disinfectant water, rinsed it with clean water after about 2 h, and let it air-dry for 1 week. After cleaning the chicken house, we used a high-pressure water gun to wash the cement floor and walls, disinfected it with 3% sodium hydroxide, and, finally, rinsed it with a high-pressure water gun, then let the chicken house air-dry for 1 week. After 1 week, the chicken house was recovered, and the chicken cages were moved into the chicken house. After closing the chicken house, we sprinkled water in the house and sprayed water on the walls at the same time, striving to achieve a relative humidity of about 75%. While humidifying the chicken house, the temperature should also be raised. Try to reach a temperature of about 25 °C in the chicken house at noon. At the same time, formalin and potassium permanganate were used for fumigation. Formalin 42 mL and potassium permanganate 21 g were used per cubic meter. The specific operation is to first pour formalin into a porcelain basin and then pour potassium permanganate. At the same time, the chicken house is sealed. After about 24 h, the doors and windows of the chicken house are opened, and the plastic film is lifted for ventilation. After 3 days, the chicken house is sealed again, and the temperature of the chicken house is raised 1 day before the chicks are placed inside, and the next round of breeding is carried out. From brooding to market, the chickens are disinfected every 3 days within 42 days. If the broilers are sick, they should be disinfected once a day. We eliminated mosquitoes, flies, and insects 1 to 2 times a week.

We developed immunization programs based on the local epidemic characteristics of chicken diseases and administered various vaccines in a timely manner as required. The vaccines selected should come from regular manufacturers or imported vaccines approved by the national competent authorities and should be stored and used correctly. The vaccine schedule is shown in Table 1.

### 2.3. Animal Environmental Parameters

We adjusted the ambient temperature according to the age of the flock (see Table 2). Lighting is an important factor affecting broiler feed intake, growth, and mortality. We used incandescent lamps and illuminated the flock by alternating between continuous light and short periods of darkness during the entire breeding period, following the principle of light intensity from strong to weak. The illumination time and illumination intensity are shown in Table 3. The ventilation standard we used was 0.85 m^3^/h per kilogram of body weight. For humidity control of the chicken house, see Table 4.

### 2.4. Eperimental Design

**Figure 1:**
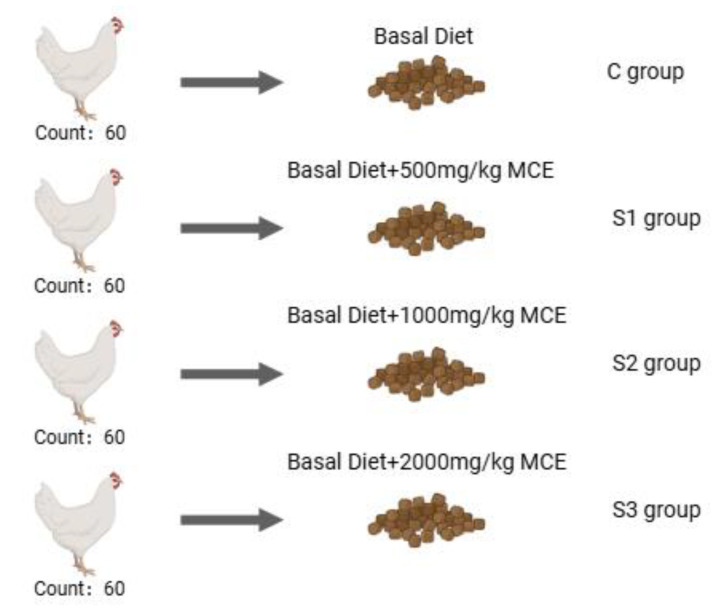
Experimental grouping of broiler chickens. This figure illustrates the randomized allocation of 240 Arbor Acres (AA) broiler chicks into four treatment groups, with each group containing six replicates of 10 chicks each. The dosing levels of M. chinensis extract for each group were based on established experimental protocols. The design ensures a balanced distribution of subjects to examine the effects on growth performance, serum biochemistry, antioxidant capacity, immune function, and gut microbiota over a 42-day period.

This study employed a single-factor randomized block design. A total of 240 healthy, 1-day-old AA broiler chickens of uniform weight (equal numbers of males and females) were randomly allocated to four treatment groups, each with six replicates and 10 chickens per replicate. The experiment lasted 42 days, divided into a starter phase (days 1–21) and a grower phase (days 22–42). Dose levels were set based on studies by Wang and Zhang [19,20], and the experimental groups are illustrated in Figure 1.

### 2.5. Diet Composition

The basal diet was formulated according to the NRC (1994) nutritional requirements for broilers and the Chinese feeding standards for chickens, adjusted based on practical production needs [21]. The diet composition and nutritional levels are provided in Table 5.

### 2.6. Growth Performance, Organ Index, and Serum Biochemical Analysis

The 42-day experiment involved fasting broilers for 12 h (with free access to water) on days 1, 21, and 42, and then weighing them to record weight change (to the nearest 0.01). Each broiler was weighed individually. The scale type is an electronic counting scale, model TSC, produced by Shenzhen Feiya Scale Co., Ltd. (Shenzhen, China). Daily feed intake and mortality were also recorded. Average daily gain (ADG), average daily feed intake (ADFI), and feed conversion ratio (FCR) were calculated. Clean drinking water was provided 24 h a day and mashed feed was available ad libitum throughout the experiment.

Following dissection, the cardiac, liver, spleen, lungs, Renals, and Bursa of Fabricius were collected and cleaned of adipose tissue. The organs were weighed after blotting blood from their surfaces with filter paper, and their indices were calculated based on the body weight ratio using the formula:

Organ Index (g/kg) = Organ Fresh Weight/Live Body Weight.

On day 42, blood samples were obtained from eight randomly selected chickens per group, chosen for their average body weight. Blood was collected in tubes and left at room temperature for 2 h, then centrifuged at 3000 rpm for 10 min at 4 °C. Serum was divided into four sterile tubes and stored at −80 °C, with one sample used for serum biochemical analysis and the remaining three for immune and antioxidant assessments.

Serum biochemical indicators included creatine kinase (CK), lactate dehydrogenase (LDH), alanine aminotransferase (ALT), aspartate aminotransferase (AST), alkaline phosphatase (ALP), total protein (TP), albumin (ALB), globulin (GLB), uric acid (UA), triglycerides (TG), low-density lipoprotein cholesterol (LDL-C), high-density lipoprotein cholesterol (HDL-C), total cholesterol (TC), and glucose (GLU). All assay kits were obtained from Shanghai Kehua Bio-Engineering Co., Ltd. (Shanghai, China).

### 2.7. Sample Collection

#### 2.7.1. Serum Collection

On day 42 of the experiment, blood samples were collected from eight randomly selected chickens per group with average body weights. Blood was drawn into collection tubes and left to stand at room temperature for 2 h, then centrifuged at 3000 rpm for 10 min at 4 °C. The serum was aliquoted into four sterile centrifuge tubes, sealed, and stored at −80 °C. One aliquot was designated for serum biochemical analysis, while the remaining three were reserved for immune and antioxidant assessments.

#### 2.7.2. Liver Sampling

On day 42, 8 chickens in each group were randomly selected based on their average body weight for liver samples. After the chickens were euthanized, liver samples were collected by cutting three evenly sized sections from the junction of the two lobes of the liver of each chicken. Each sample was gently rinsed with physiological saline, blotted dry with filter paper, and placed into cryogenic vials. The samples were immediately frozen in liquid nitrogen, then transferred to a −80 °C freezer for preservation, pending analysis of antioxidant and related biochemical parameters, including total antioxidant capacity (T-AOC), glutathione peroxidase (GSH-PX), superoxide dismutase (SOD), catalase (CAT), and malondialdehyde (MDA).

#### 2.7.3. Liver Homogenate Preparation

Under low-temperature conditions, a precise amount of liver tissue was carefully dissected from the liver organ, weighed, and placed in a 2 mL grinding tube. Tissue lysis buffer (in a 1:9 tissue-to-buffer volume ratio) from a specified assay kit was added, along with grinding beads. The sample was homogenized using a tissue grinder at 4 °C with a program of 60 Hz for 30 s, followed by a 30-s pause, repeated for three cycles. The homogenate was centrifuged at 3000 rpm for 10 min, and the supernatant was transferred to sterile, nuclease-free tubes. Samples were stored at −80 °C and analyzed within one week.

#### 2.7.4. Intestinal and Luminal Content Sampling

After dissection, the contents of the small intestine (duodenum, jejunum, and ileum) and large intestine (cecum and colon) were collected and homogenized. The small intestine contents were a mixture of the contents from the duodenum, jejunum, and ileum, while the large intestine contents were a mixture of the contents from the cecum and colon. The homogenized samples were divided into three equal portions for subsequent analysis. Additionally, the entire duodenum and cecum segments were excised, rinsed with physiological saline, blotted dry with filter paper, and placed into 2 mL low-temperature tubes. The samples were rapidly frozen in liquid nitrogen and stored at −80 °C for further analysis of tight junction protein expression in the intestinal tissues.

### 2.8. Liver and Serum Antioxidant Assays

After liver homogenization, the protein concentration in the supernatant was determined using a BCA Protein Assay Kit. Following the manufacturer’s protocol (Boxbio), activities of total antioxidant capacity (T-AOC), superoxide dismutase (SOD), catalase (CAT), and glutathione peroxidase (GSH-PX) were measured, along with malondialdehyde (MDA) levels in liver homogenates. GSH-PX, nitric oxide (NO) activity and MDA levels in serum were similarly measured according to the kit instructions.

### 2.9. Immune Performance Assays

The serum concentrations of immunoglobulins (IgA, IgM, and IgG) and cytokines (IL-4, IL-10, and IFN-γ) were measured according to the instructions provided by Shanghai Enzyme-linked Biotechnology Co., Ltd. (Shanghai, China), using standard curves to quantify each analyte.

### 2.10. Intestinal Barrier Gene Analysis

Frozen samples of the duodenum and cecum organs were used to extract total RNA via the TRIzol method, followed by reverse transcription to synthesize cDNA. The reverse transcription reaction conditions were as follows: 37 °C for 15 min, 85 °C for 5 s, and 4 °C indefinitely. Quantitative PCR was performed with an initial denaturation at 95 °C for 30 s, followed by 45 cycles of denaturation at 95 °C for 5 s and annealing/extension at 60 °C for 30 s. Gene expression was normalized using β-actin as the internal reference and quantified using the comparative cycle threshold method (2^−ΔΔCt^). Primer sequences are listed in Table 6.

### 2.11. Short-Chain Fatty Acid (SCFA) Analysis in Intestinal Contents

Quantitative analysis of short-chain fatty acids (SCFAs), including acetic acid, propionic acid, butyric acid, and valeric acid, was performed on the contents from both small and large sections of the intestine using high-performance gas chromatography. A 1.0 g sample of intestinal content was placed in a 10 mL sterile centrifuge tube, mixed with 5 mL of ultrapure water, and vortexed for 30 min. The sample was then stored overnight at 4 °C. After centrifugation at 10,000 rpm for 10 min, the supernatant was collected. An additional 4 mL of ultrapure water was added to the precipitate, mixed again for 30 min, and centrifuged to obtain a second supernatant, which was then combined with the first. The combined supernatant was centrifuged at 12,000 rpm for 15 min. Then, 900 μL of the final supernatant was combined with 100 μL of 25% metaphosphoric acid in a 2 mL centrifuge tube. After mixing, the solution was left at room temperature for 3–4 h, followed by high-speed centrifugation. The resulting supernatant was filtered through a 0.45 μm nylon membrane before injection.

### 2.12. 16S rRNA Microbial Sequencing of Intestinal Contents

#### 2.12.1. DNA Extraction and PCR Amplification

To assess changes in the intestinal microbiota of broilers, 16S rRNA amplicon sequencing was performed on samples from both the small and large intestinal contents. DNA was extracted from these microbiota samples, and the genomic DNA obtained was quantified. PCR amplification was conducted on the V3–V4 variable regions of the 16S rRNA gene using the primers 338F (5’-ACTCCTACGGGAGGCAGCAG-3’) and 806R (5’-GGACTACHVGGGTWTCTAAT-3’).

#### 2.12.2. Illumina MiSeq Sequencing

PCR products were recovered using a 2% agarose gel, purified, eluted in Tris-HCl, and checked with 2% agarose electrophoresis. Quantification was conducted using QuantiFluorM-ST (Promega, USA). Libraries were constructed for paired-end 2 × 300 sequencing and sequenced using the Illumina MiSeq PE300 platform.

#### 2.12.3. Data Processing

Bacterial diversity indices (Sobs, Chao, Ace, and Shannon indices) were determined based on operational taxonomic units (OTUs) using Mothur (v.1.30.2, http://mothur.org, accessed on 1 September 2024). Clustering was performed with the USEARCH11-uparse algorithm, and taxonomy was assigned using the SILVA 138/16S_bacteria database. R software (v.4.1.0) was used for principal component analysis (PCA), principal coordinates analysis (PCoA), species composition analysis, and differential analysis. Distribution characteristics and sample similarity among groups were examined at the phylum and family levels, and differences among the top 50 genera were assessed using the Kruskal–Wallis rank-sum test. Correlation analysis was performed on the top 50 genera using Spearman’s method. All analyses were processed through the Meiji Cloud Platform. Comparisons with the control group (C) were considered statistically significant at * *p* < 0.05, ** *p* < 0.01, and *** *p* < 0.001.

### 2.13. Statistical Analysis

Data from this experiment were initially organized using Excel, followed by statistical analysis with GraphPad Prism 9.0 (GraphPad Software, San Diego, CA, USA) and SPSS 25.0. A one-way analysis of variance (ANOVA) was used to evaluate the significance of differences across multiple groups, using Duncan’s multiple range test for post hoc comparisons. Results are expressed as mean ± standard error of the mean (SEM). Comparisons with the control group were considered non-significant at *p* > 0.05, significant at * *p* < 0.05, and highly significant at ** *p* < 0.01.

## 3. Results

### 3.1. Growth Performance, Organ Index, and Serum Biochemical Indicators

From days 1 to 21, there were no significant differences in ADFI and ADG between C and the MCE-supplemented groups (*p* > 0.05). However, the F/G ratio of the S2 group was significantly higher than that of the other three groups (*p* < 0.05). From days 22 to 42, the ADG of the S3 group was significantly higher than that of the C (*p* < 0.05). Although no significant difference in F/G was observed, the result was lower in the S3 group compared to the C group (*p* > 0.05). Overall, from days 1 to 42, the ADG of the S3 group was significantly improved (*p* < 0.05), while the F/G ratio was similar to the results observed from days 22 to 42 in Table 7.

As shown in Table 8, the renal index increased significantly in the S2 and S3 groups compared to the control group (*p* < 0.05). The thymus index increased significantly in both the S1 and S3 groups (*p* < 0.01), while there were no significant differences in cardiac, liver, spleen, or lung indices (*p* > 0.05).

The data in Table 9 indicate that MCE supplementation had significant effects on the serum biochemical indicators of broiler chickens. For liver function-related indicators, AST levels in the S2 group decreased to 344.16 U/L (*p* = 0.06), approaching significance), while ALT levels were significantly reduced in all MCE-supplemented groups, with the lowest level observed in the S2 group (*p* < 0.01). LDH levels in the S2 group also showed a significant reduction (*p* < 0.01), indicating reduced tissue damage and improved cellular metabolism. In terms of lipid metabolism, TG levels significantly decreased in the MCE-supplemented groups (*p* < 0.01), with the S2 group showing the lowest value. HDL-C levels were significantly elevated in the S2 and S3 groups (*p* < 0.01), while LDL-C levels in the S3 group were the lowest, although the difference was not statistically significant (*p* > 0.05). Other indicators, such as uric acid UA, GLU, TP, and ALB, showed no significant differences among the groups (*p* > 0.05), although the UA level in the S3 group was the lowest.

Multiple comparison results indicated that MCE supplementation significantly increased HDL-C concentration (*p* < 0.01) and showed a trend toward lower LDL-C in the S1 group (*p* < 0.05). Additionally, MCE supplementation decreased serum TC concentration, though this reduction was not statistically significant (*p* > 0.05). Serum TG levels were significantly lower in the S1 and S2 groups (*p* < 0.05). For GLU concentration, only the S2 group showed a significant increase (*p* < 0.05).

### 3.2. Antioxidant Index Analysis in Liver and Serum

**Figure 2:**
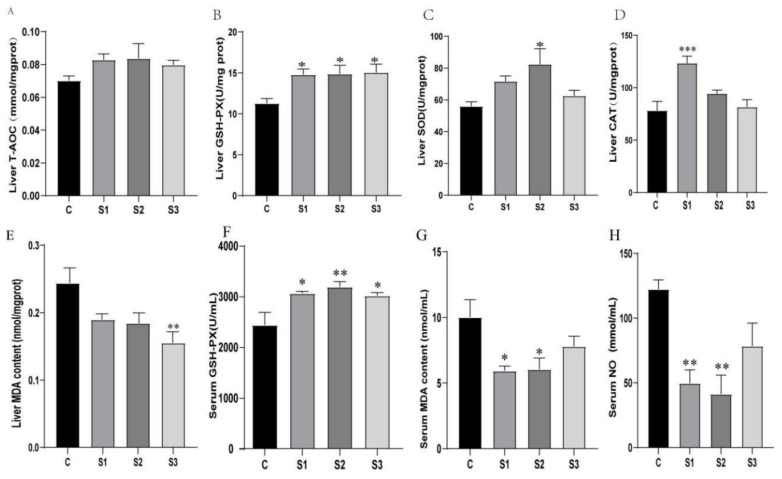
Effects of M. chinensis extract on antioxidant activity in serum and liver of white feather broilers: (**A**) total antioxidant capacity (T-AOC) in the liver; (**B**) glutathione peroxidase (GSH-PX) activity in the liver; (**C**) superoxide dismutase (SOD) activity in the liver; (**D**) catalase (CAT) activity in the liver; (**E**) malondialdehyde (MDA) levels in the liver; (**F**) glutathione peroxidase (GSH-PX) activity in the serum; (**G**) malondialdehyde (MDA) levels in the serum; (**H**) nitric oxide (NO) levels in the serum. * *p* < 0.05, ** *p* < 0.01, *** *p* < 0.001.

Total antioxidant capacity (T-AOC) reflects the collective effect of antioxidants and antioxidant enzymes, offering protection against oxidative stress induced by reactive oxygen species. The results of the antioxidant capacity analysis of M. chinensis extract (MCE) on white feather broilers are shown in Figure 2. Compared to the control group (C), MCE-supplemented groups (S1, S2, and S3) demonstrated an upward trend in hepatic T-AOC, though this increase was not statistically significant (*p* > 0.05).

Following MCE supplementation, glutathione peroxidase (GSH-PX) activity significantly increased in both liver and serum (*p* < 0.05), with the S2 group showing a highly significant elevation in serum GSH-PX activity (*p* < 0.01). Superoxide dismutase (SOD) activity in the liver also showed improvement, with a statistically significant increase observed in the S2 group (*p* < 0.05). Malondialdehyde (MDA) levels, an indicator of lipid peroxidation, were reduced in both liver and serum across MCE-treated groups. In liver tissue, only the S3 group showed a statistically significant reduction in MDA levels (*p* < 0.05), while serum MDA levels were significantly lower in both the S1 and S2 groups (*p* < 0.05).

Additionally, catalase (CAT) activity in the liver was significantly elevated in the S1 group compared to the control (*p* < 0.05), with moderate, though non-significant, increases in the S2 and S3 groups (*p* > 0.05). Nitric oxide (NO) levels, representing a highly reactive vasodilative free radical, decreased in all MCE-treated groups. The S1 and S2 groups showed a highly significant reduction in serum NO levels (*p* < 0.01), while the S3 group exhibited a downward trend, though without statistical significance (*p* > 0.05).

### 3.3. Immune Performance Analysis

**Figure 3:**
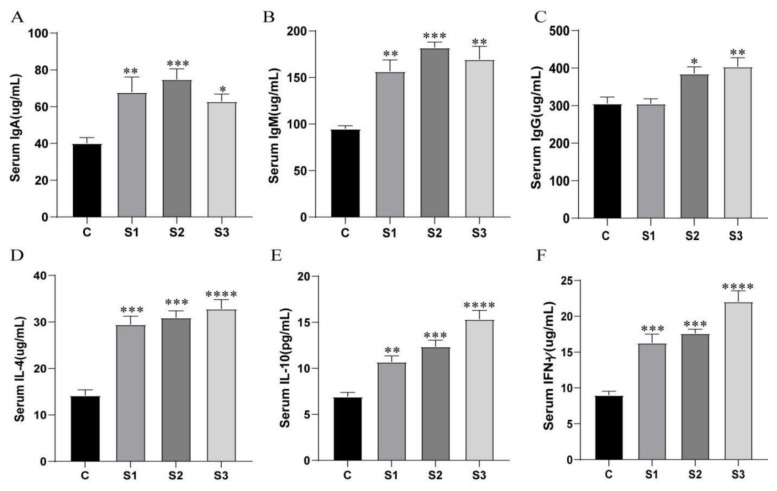
Effects of M. chinensis extract on the immune performance of white feather broilers: (**A**) serum IgA levels; (**B**) serum IgM levels; (**C**) serum IgG levels; (**D**) serum IL-4 levels; (**E**) serum IL-10 levels; (**F**) serum IFN-γ levels * *p* < 0.05, ** *p* < 0.01, *** *p* < 0.001, **** *p* < 0.0001.

The impact of varying doses of M. chinensis extract on the immune performance of white feather broilers was examined, with results presented in Figure 3. As shown, compared to the control group (C), dietary supplementation with M. chinensis extract significantly or highly significantly increased immunoglobulin IgA and IgM levels (*p* < 0.05). For immunoglobulin IgG, levels in the S1 group were comparable to the control, while the S2 group exhibited a significantly higher IgG concentration in serum compared to the control (*p* < 0.05), and the S3 group showed an even greater increase (*p* < 0.01).

In terms of cytokines IL-4, IL-10, and IFN-γ, all groups supplemented with M. chinensis extract demonstrated consistently elevated levels. Each of these cytokines (IL-4, IL-10, and IFN-γ) was significantly increased (*p* < 0.01), with the cytokine levels rising in a dose-dependent manner as the concentration of M. chinensis extract increased.

### 3.4. Intestinal Barrier Gene Expression Analysis

**Figure 4:**
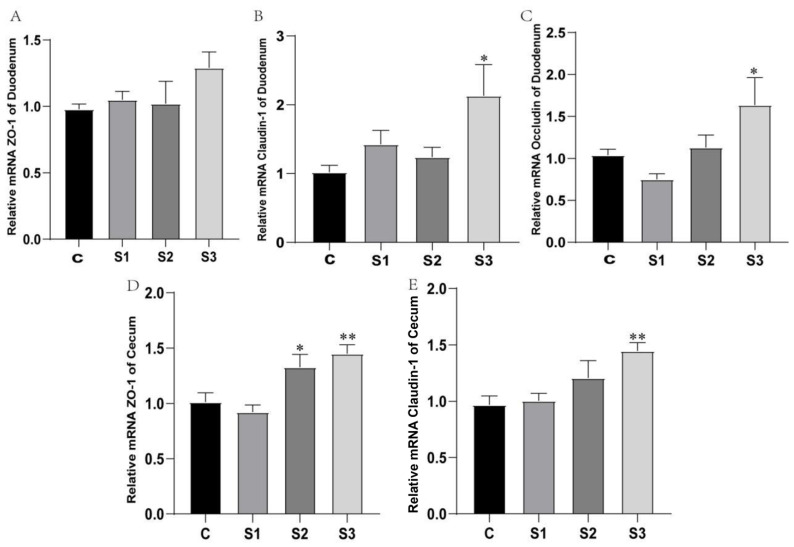
Effects of MCE on intestinal tight junction protein gene expression in white feather broilers: (**A**) ZO-1 expression in the duodenum; (**B**) Claudin-1 expression in the duodenum; (**C**) Occludin expression in the duodenum; (**D**) ZO-1 expression in the cecum; (**E**) Claudin-1 expression in the cecum. * *p* < 0.05, ** *p* < 0.01.

The small intestine is primarily responsible for digestion, while the large intestine mainly facilitates absorption. Given the highly developed cecum in chickens, we selected the duodenum from the small intestine and the cecum from the large intestine to examine the gene expression of tight junction proteins. This analysis aimed to preliminarily assess whether dietary supplementation with MCE has a protective effect on the intestinal barrier of white feather broilers. The results are shown in Figure 4.

In the duodenum, the expression levels of ZO-1 and Claudin-1 genes showed an upward trend. The expression of the two genes in the S1 and S2 groups increased, and the expression level of the Occludin gene in S2 showed an upward trend; however, they were not statistically significant (*p* > 0.05). There was no statistically significant difference in the expression level of ZO-1 in the S3 group (*p* > 0.05), while the expression levels of Claudin-1 and Occludin were significantly increased (*p* < 0.05). In the S3 group, ZO-1 expression was also not significantly different (*p* > 0.05), while the expression levels of Claudin-1 and Occludin were significantly elevated (*p* < 0.05).

In the cecum, the effects on ZO-1 and Claudin-1 gene expression are illustrated in Figure 4D,E. Notably, ZO-1 expression in the S1 group was slightly lower than in the control group, while the S2 (*p* < 0.05) and S3 (*p* < 0.01) groups showed significant increases in ZO-1 expression. Claudin-1 expression was elevated across all MCE-supplemented groups, with a positive dose-dependent trend. The S3 group displayed a highly significant increase in Claudin-1 expression (*p* < 0.01).

These preliminary results suggest that dietary supplementation with MCE at certain doses may provide a protective effect on the intestinal barrier in broilers.

### 3.5. Short-Chain Fatty Acid (SCFA) Analysis in Intestinal Contents

**Figure 5:**
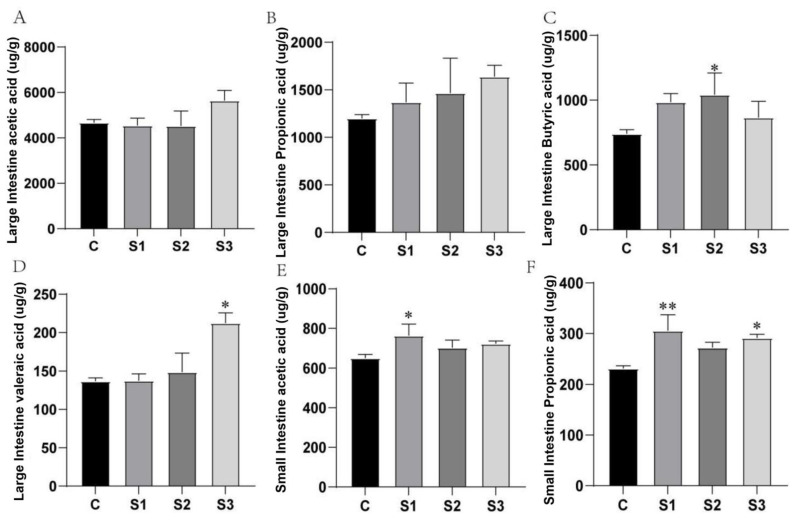
Effects of MCE on short-chain fatty acid production in intestinal contents of white feather broilers: (**A**) acetic acid in the large intestine; (**B**) propionic acid in the large intestine; (**C**) butyric acid in the large intestine; (**D**) valeric acid in the large intestine; (**E**) acetic acid in the small intestine; (**F**) propionic acid in the small intestine. * *p* < 0.05, ** *p* < 0.01.

Short-chain fatty acids (SCFAs) play roles in regulating appetite, body weight, and immunity in animals. To further explore the effects of dietary supplementation with MCE on SCFA production, the levels of acetic acid, propionic acid, butyric acid, and valeric acid were measured in the contents of the small and large intestines. As shown in Figure 5, the small intestine predominantly produced acetic acid and propionic acid, while other SCFAs were undetectable.

MCE supplementation increased the levels of acetic acid and propionic acid in the small intestine. Compared to the control group (C), the S1 group showed a significant increase in acetic acid content (*p* < 0.05) and a highly significant increase in propionic acid (*p* < 0.01). In the S3 group, propionic acid levels were also significantly higher than in the control group (*p* < 0.05). The specific results are shown in Figure 5E,F.

In the large intestine, MCE supplementation led to increased levels of acetic acid, propionic acid, and valeric acid across the S1, S2, and S3 groups, displaying a dose-dependent trend. In the S3 group, valeric acid levels in the large intestine showed a significant increase (*p* < 0.05). Although the S3 group also showed an increase in butyric acid, it was slightly lower compared to the S1 and S2 groups. The S2 group demonstrated a significant elevation in butyric acid content in the large intestine (*p* < 0.05).

### 3.6. Intestinal Microbiota Modulation

#### 3.6.1. Taxonomic Identification from Microbial Sequencing Results

**Figure 6:**
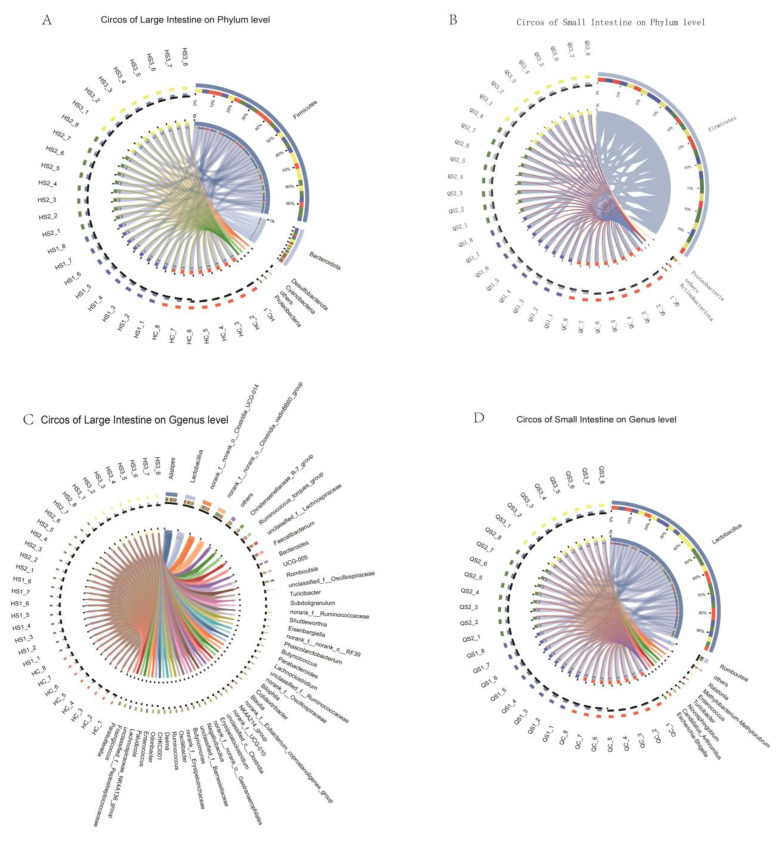
Microbial composition analysis of intestinal contents: (**A**) gate level composition analysis of colonic contents; (**B**) gate level composition analysis of small intestine contents; (**C**) analysis of the genus level composition of the contents of the large intestine; (**D**) analysis of genus level composition of small intestine contents.

After 42 days of feeding diets supplemented with three different doses of MCE, a total of 72 samples of large and small intestinal contents were collected from the control (C), S1, S2, and S3 groups of white feather broilers. Diversity analysis of these 72 samples yielded 3,576,298 optimized sequences, with an average sequence length of 418 bp. Taxonomic annotation identified 19 phyla, 155 families, 316 genera, and 571 species. At the phylum level, the top five taxa identified were Firmicutes, Bacteroidota, Proteobacteria, Desulfobacterota, and Cyanobacteria. At the genus level, the dominant genera included Lactobacillus, Alistipes, and Romboutsia. Figure 6 illustrates the taxonomic composition at the phylum level for the large (A) and small (B) intestinal contents. The large intestinal contents were primarily composed of Firmicutes, Bacteroidota, Proteobacteria, Desulfobacterota, and Cyanobacteria, while the small intestinal contents were dominated by Firmicutes, Proteobacteria, and Actinobacteria. As shown in Figure 6, microbial diversity was richer in the large intestinal contents, which exhibited a greater variety of phyla and genera compared to the small intestinal contents.

#### 3.6.2. Effects of MCE on OTU Counts in Intestinal Contents of White Feather Broilers

**Figure 7:**
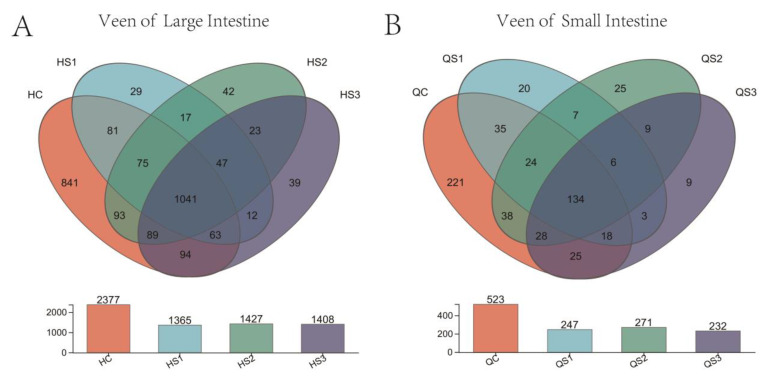
Effects of dietary MCE on OTU counts in the intestinal microbiota of broilers: (**A**) OTU count changes in large intestinal contents; (**B**) OTU count changes in small intestinal contents.

Venn diagrams are useful for displaying the number of shared and unique species at the OTU level across multiple groups or samples, providing a visual comparison of species composition similarity and overlap among different environmental samples. After removing chimeras using the UPARSE pipeline, operational taxonomic units (OTUs) were clustered at 97% sequence similarity to assess the effects of different doses of MCE on the intestinal microbiota in the small and large intestines of broilers (Figure 7).

As shown, the large intestine contained a greater number of microbial OTUs than the small intestine. Notably, across both the small and large intestines, the OTU counts in all MCE-treated groups were lower than those in the control group. In the large intestine, 1041 OTUs were shared among the control group (C) and treatment groups (S1, S2, and S3), while 134 OTUs were shared in the small intestine. These findings suggest that dietary supplementation with MCE can alter the composition and diversity of intestinal microbiota in broilers at the OTU level.

#### 3.6.3. Effects of MCE on Microbial Diversity in Small and Large Intestinal Contents

**Figure 8:**
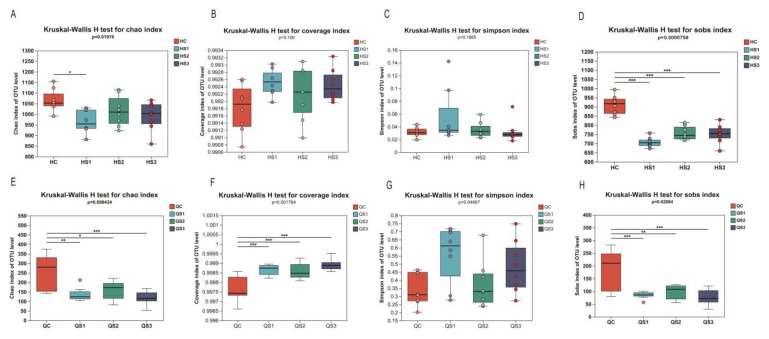
Effects of dietary MCE on alpha diversity of intestinal microbiota in broilers: (**A**) Chao index for large intestinal contents; (**B**) coverage index for large intestinal contents; (**C**) Simpson index for large intestinal contents; (**D**) Sobs index for large intestinal contents; (**E**) Chao index for small intestinal contents; (**F**) coverage index for small intestinal contents; (**G**) Simpson index for small intestinal contents; (**H**) Sobs index for small intestinal contents. * *p* < 0.05, ** *p* < 0.01, *** *p* < 0.001.

Alpha diversity reflects the microbial diversity within a specific area or ecosystem, often measured using indices of community richness (e.g., Chao, Sobs, Ace), community diversity (e.g., Shannon, Simpson), and community coverage (e.g., coverage). The effects of MCE on the richness, coverage, diversity, and evenness of intestinal microbiota in broilers are shown in Figure 8.

In the large intestine, the Chao index decreased across the S1, S2, and S3 groups compared to the control group (C), with a significant reduction observed in the S1 group (*p* < 0.05, Figure 8A). The coverage index increased in all treatment groups, though not significantly (*p* = 0.106, Figure 8B). The Simpson index showed no significant changes in OTU diversity at the large intestinal level (*p* = 0.188, Figure 8C). However, the Sobs index in the large intestine was significantly reduced in all treatment groups compared to the control (*p* < 0.01, Figure 8D). These findings indicate that supplementation with MCE reduced microbial richness in the large intestine but showed a trend toward higher diversity and coverage.

In the small intestine, MCE similarly impacted microbial diversity. The Chao index showed a significant reduction across the S1, S2, and S3 groups (*p* = 0.008, Figure 8E), and the coverage index demonstrated a significant increase (*p* < 0.001, Figure 8F). The Simpson index exhibited significant changes (*p* = 0.048), though without a dose-dependent effect. The Sobs index was significantly lower across all treatment groups (*p* < 0.01), consistent with the Chao index results. These findings suggest that MCE reduced microbial richness in the small intestine while increasing microbial diversity and coverage, paralleling the effects observed in the large intestine.

**Figure 9:**
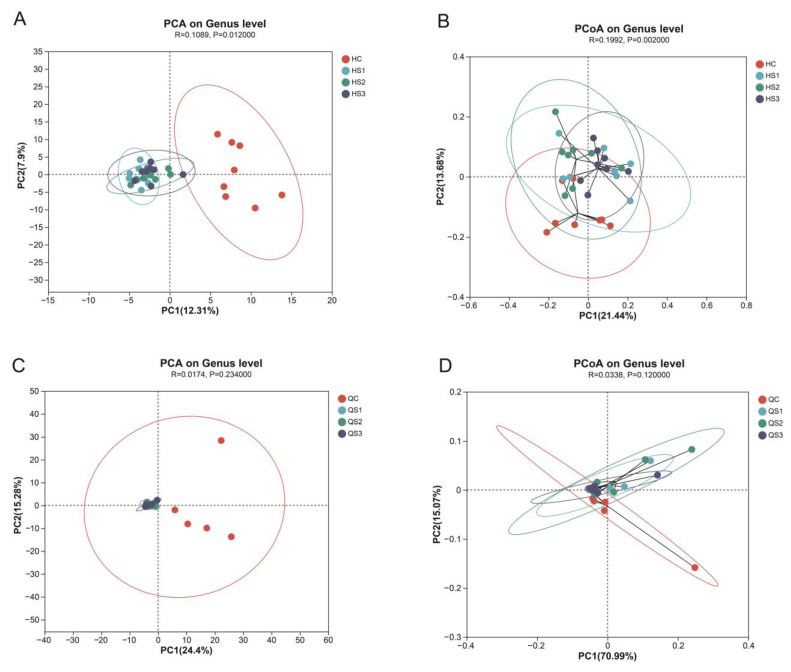
Principal component analysis of intestinal microbiota at the genus level in broilers supplemented with MCE: (**A**) PCA plot of large intestinal microbiota; (**B**) PCoA plot of large intestinal microbiota; (**C**) PCA plot of small intestinal microbiota; (**D**) PCoA plot of small intestinal microbiota.

Beta diversity analysis compares species diversity across different habitats or microbial communities to examine community composition similarities or differences among groups. The effects of MCE on beta diversity in broiler intestinal microbiota are shown in Figure 9. For large intestinal contents, as illustrated in Figure 9A,B, PCA analysis indicated that the microbial communities in the S1, S2, and S3 groups formed distinct clusters at the genus level, with significant separation from the control group (C). Principal Component 1 (PC1) and Principal Component 2 (PC2) accounted for 12.31% and 7.9% of the variance, respectively, with significant Euclidean distance differences between groups (*p* < 0.05). This suggests that MCE intervention altered microbial diversity in the large intestine (Figure 9A). Similarly, PCoA analysis based on Bray–Curtis distance (Figure 9B) revealed highly significant differences between treatment and control groups (*p* < 0.01), consistent with the PCA findings.

In contrast, Figure 9C,D illustrates the effects of MCE on small intestinal microbiota, showing no significant differences in either Euclidean or Bray–Curtis distances compared to the control group (*p* > 0.05).

In summary, dietary supplementation with MCE demonstrated a regulatory effect on broiler intestinal microbiota diversity, primarily affecting the large intestine.

#### 3.6.4. Effects of Dietary MCE on Intestinal Microbiota Composition and Differential Analysis in White Feather Broilers

**Figure 10:**
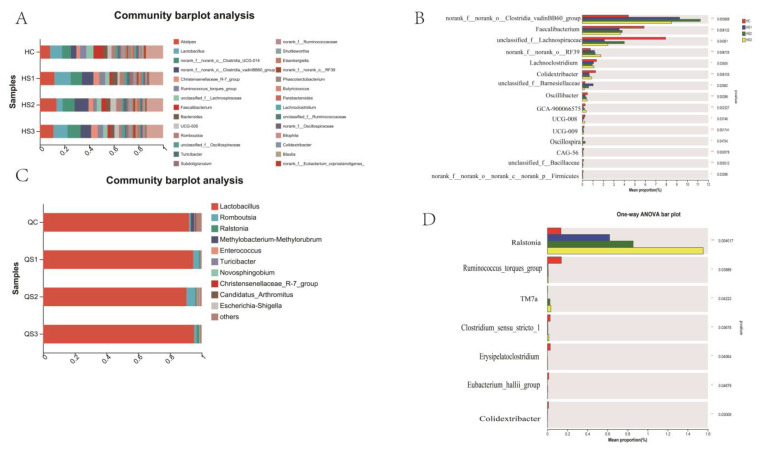
Differential analysis of intestinal microbiota at the genus level in broilers supplemented with MCE: (**A**) bar chart of the large intestinal microbial community composition; (**B**) inter-group differential analysis of large intestinal microbiota; (**C**) bar chart of the small intestinal microbial community composition; (**D**) inter-group differential analysis of small intestinal microbiota. * *p* < 0.05, ** *p* < 0.01.

To further analyze the regulatory effects of MCE on intestinal microbiota at the genus level, the composition and differential analysis of the microbiota in broilers supplemented with MCE are shown in Figure 10. In the large intestine, predominant genera included Alistipes, Lactobacillus, norank_f__norank_o__Clostridia_UCG-01, norank_f__norank_o__Clostridia_vadinBB60_group, and Christensenellaceae_R-7_group (Figure 10A). Among these, the relative abundance of norank_f__norank_o__Clostridia_vadinBB60_group and norank_f__norank_o__RF39 was significantly elevated in the S1, S2, and S3 groups (*p* < 0.01), while the abundance of Faecalibacterium and Lachnospiraceae decreased in the large intestine of the treatment groups (*p* < 0.01).

In the small intestine, more than 80% of the microbial community consisted of Lactobacillus, followed by Romboutsia and Ralstonia. Notably, the genus Methylobacterium-Methylorubrum was more abundant in the control group than in the extract-treated groups (Figure 10C). The genus Ralstonia was significantly increased in the S1, S2, and S3 groups (*p* < 0.01, Figure 10D).

These findings suggest that dietary supplementation with MCE can modulate the intestinal microbiota composition in broilers, influencing both small and large intestinal microbial communities.

#### 3.6.5. Correlation Analysis Between Short-Chain Fatty Acids and Intestinal Microbiota

**Figure 11:**
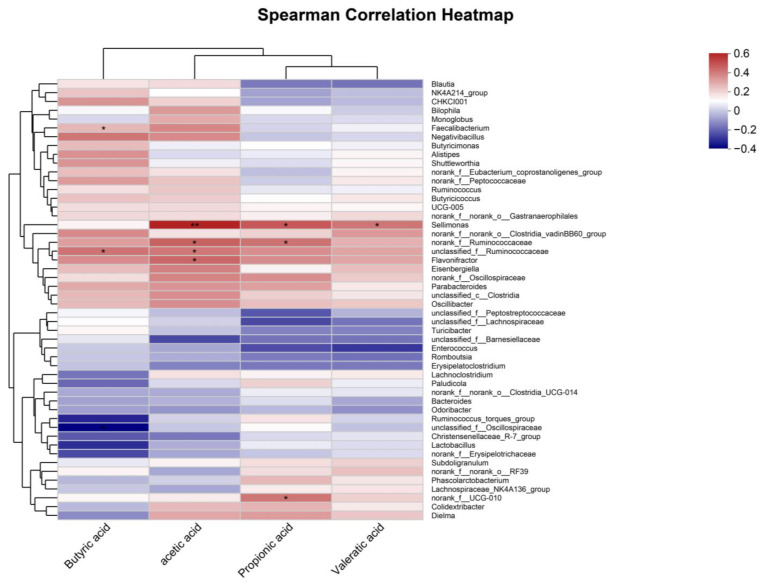
Correlation analysis between short-chain fatty acids and microbiota in the large intestine. This figure presents the Spearman correlation analysis between short-chain fatty acids (SCFAs) and the microbial genera in the large intestine. * *p* < 0.05, ** *p* < 0.01.

Based on the short-chain fatty acid (SCFA) detection results in this study, a correlation analysis was performed on the large intestinal microbiota, as shown in Figure 11. Spearman correlation analysis was conducted between the top 50 genera by relative abundance and the SCFAs in the intestinal contents. In the large intestinal microbiota, acetic acid showed positive correlations with norank_f__Ruminococcaceae, unclassified_f__Ruminococcaceae, Flavonifractor, and Sellimonas. Propionic acid was primarily associated with Sellimonas, norank_f__UCG-010, and norank_f__Ruminococcaceae. Butyric acid displayed positive correlations with unclassified_f__Ruminococcaceae and Faecalibacterium, while valeric acid was mainly correlated with Sellimonas. These genera, which are positively associated with SCFA production, contribute to intestinal health and play an active role in maintaining a healthy gut environment.

## 4. Discussion

*Mosla chinensis Maxim.* has a complex composition and is utilized both as a medicinal treatment and as a food source [22]. It contains abundant nutrients and exhibits pharmacological effects, yet its application in the livestock industry remains underexplored. Growth performance is one of the most straightforward indicators of the nutritional and economic efficacy of plant-based feed additives [23]. Studies by Yang et al. [24] demonstrated that adding aqueous Trigonella foenum-graecum (fenugreek) seed extract to the diet significantly improved the average daily gain and feed conversion ratio in broilers. Similarly, research by Enas Toson et al. [25] indicated that incorporating Glycyrrhiza methanolic extract at 3 g/kg in broiler diets markedly enhanced body weight and feed efficiency. These findings suggest that both volatile and non-volatile components of plant-derived extracts can positively influence broiler growth performance.

In this study, during the 1–21 day phase, there were no significant differences in average daily feed intake (ADFI) and average daily gain (ADG) between C and the MCE-supplemented groups. However, the feed-to-gain ratio (F/G) of the S2 group was significantly higher than that of the other three groups, indicating a lower feed efficiency in the S2 group. This may be due to the interference of certain active components in MCE with the digestion and absorption processes in chicks, or it could be related to the adaptation issues associated with the chicks’ early physiological condition [26]. During the 22–42 day phase and the entire 1–42 day period, the significant improvement in ADG in the S3 group further demonstrated that the addition of MCE promotes the growth of broiler chickens. At the same time, J. A. Omar et al. [27] reported that adding Natural Herb Extract to broiler feed significantly improved body weight, feed-to-gain ratio, and reduced mortality. These results suggest that MCE is a promising plant-based feed additive that can effectively enhance the production performance and economic benefits of broiler chickens.

MCE supplementation significantly affected liver function and lipid metabolism in broiler chickens. Regarding liver function, AST levels in the S2 group decreased to 344.16 U/L (*p* = 0.06), and both ALT and LDH levels were significantly reduced in all MCE-supplemented groups (*p* < 0.01), suggesting that MCE may improve liver health by alleviating tissue damage and enhancing cellular metabolism. MCE supplementation significantly reduced triglyceride (TG) levels (*p* < 0.01), with the lowest TG value observed in the S2 group, while it increased HDL-C levels in the S2 and S3 groups (*p* < 0.01) and decreased LDL-C levels in the S3 group (although not significant, *p* > 0.05). These results indicate that MCE may improve lipid metabolism by regulating lipid synthesis and breakdown, although further research is needed. In some studies on feed additives for broiler chickens, the addition of plant extract mixtures to the feed significantly improved growth performance but had no significant effect on serum lipid parameters [28].

In this study, the addition of MCE significantly increased the levels of glutathione peroxidase (GSH-PX), catalase (CAT), total antioxidant capacity (T-AOC), and superoxide dismutase (SOD) in both serum and liver, while controlling the levels of malondialdehyde (MDA) and nitric oxide (NO), indicating that MCE supplementation enhanced the antioxidant capacity of broilers and may have a direct impact on their health and productivity. The increase in antioxidant enzyme levels suggests an enhanced cellular defense against oxidative stress, helping to protect cells from oxidative damage, particularly in key tissues involved in metabolism and immune responses, thus improving immune function and disease resistance, reducing disease incidence, and increasing survival rates. At the same time, the reduction in MDA levels indicates effective inhibition of lipid peroxidation, reducing oxidative damage and protecting cell membranes and tissues, preventing cellular dysfunction and inflammation caused by lipid oxidation, thereby promoting healthy growth and higher productivity. Moreover, the regulation of NO levels suggests that MCE could reduce inflammation, further improving feed conversion rate (FCR) and growth rate. Therefore, this study demonstrates that MCE, by enhancing antioxidant defense mechanisms, reducing oxidative damage, and minimizing inflammation, not only improved the health of broilers but also increased their productivity. These results are consistent with existing literature, which indicates that enhancing antioxidant defenses has a significant positive impact on poultry health and productivity [29], thus the antioxidant effects of MCE provide an effective strategy for improving broiler growth performance.

Serum immunoglobulin levels and lysozyme activity are critical indicators of non-specific immune status in animals [30]. IL-4 is a multifunctional cytokine playing a key role in various immune responses, including Th2-mediated immunity, IgE class switching in B cells, and M2 phenotype activation in macrophages [31]. Interleukin-10 (IL-10) is an anti-inflammatory cytokine that limits immune responses to pathogens, preventing host damage; moreover, it plays a core role in infection control as a broad-spectrum, anti-inflammatory agent [32,33]. In this study, supplementation with MCE increased serum levels of IgG, IgA, and IgM in broilers and significantly elevated IL-4, IL-10, and IFN-γ levels (*p* < 0.01). Few studies have investigated the immunoenhancing effects of MCE in poultry; however, prior research shows it regulates immune activity in mice by inhibiting MAPK activation, thereby reducing NO, COX-2, TNF-α, IL-6, and IL-1β production [19]. Zhang et al. [20] also demonstrated that Elsholtzia flavonoids enhance the immune response against H1N1 in pneumonia-model mice by upregulating IL-6, TNF-α, and IFN-γ. Combined with serum biochemical results, these results further emphasize that MCE can improve the disease resistance of broiler chickens.

The intestinal barrier is the first line of defense against pathogens, crucial for maintaining gut homeostasis and protecting intestinal health [34]. Comprising an epithelial cell layer, mucosal chemical barriers, and various immune cells, the gut barrier includes tight junction proteins such as Occludin, Claudins, and ZO-1, which are essential for preserving epithelial integrity [35]. Occludin modulates epithelial permeability [36], Claudin proteins regulate paracellular transport [37], and ZO-1, located on the cytoplasmic side of cell membranes, connects tight junctions (TJs) to the actin cytoskeleton [38]. Studies have shown that dietary supplementation with ginsenoside can increase Occludin, Claudins, and ZO-1 expression in broiler intestines, enhancing epithelial integrity [39]. Similarly, Qiu et al. [40] observed statistically significant increases in Occludin, Claudin-1, and ZO-1 expression when supplementing broiler diets with Bacillus subtilis as an antibiotic alternative. In this study, the addition of MCE to broiler diets showed a comparable protective effect on intestinal integrity in the duodenum and cecum, with increased efficacy at higher doses (*p* < 0.05 in S3 compared to control). This effect may be due to bioactive compounds in Elsholtzia ciliata that act as inhibitors, reducing pathogen adhesion to epithelial or mucosal layers [41,42,43]. Additionally, compounds such as rosmarinic acid and flavonoids in Elsholtzia ciliata may help modulate gut microbiota, enhance beneficial bacteria, and stimulate volatile fatty acid production, thereby supporting gut integrity [44]. We infer that MCE may improve gut barrier function in broilers, thereby enhancing overall gut health.

Intestinal microbiota plays a crucial role in various aspects of animal health, including immunity, metabolism, and growth promotion [45,46]. Microbial fermentation produces short-chain fatty acids (SCFAs) such as acetic, propionic, butyric, and valeric acids, which indirectly affect physiological processes and may help improve immunity or prevent disease [47,48]. SCFAs stimulate specific membrane-bound receptors to regulate gut motility, hormone secretion, epithelial barrier maintenance, and immune cell function [49]. To date, no studies have explored the effects of MCE on broiler gut microbiota and SCFA production. In this study, MCE increased acetic acid (S1, *p* < 0.05) and propionic acid (S1 and S3, *p* < 0.05) levels in the small intestine and acetic, propionic, butyric (S2, *p* < 0.05), and valeric acid (S3, *p* < 0.05) levels in the large intestine, though without a clear dose-dependent trend. The dominant genera in the small intestine included Lactobacillus, Romboutsia, and Ralstonia, while Alistipes and Lactobacillus were predominant in the large intestine, wherein they play vital roles in energy production and metabolism. Although the addition of MCE did not significantly improve the average daily feed intake, average daily weight gain, and feed-to-weight ratio in the 1–21 day period, the S3 group showed a significant increase in average daily weight gain (*p* < 0.05) during the 22–42 day and overall 1–42 day periods, with a dose-dependent effect observed. We believe that MCE supplementation significantly promotes broiler growth performance in the later stages, which is closely related to improvements in gut health. Relevant studies have shown that SCFAs play a crucial role in gut health and growth performance in animals [50]. SCFAs promote the proliferation and repair of intestinal epithelial cells, enhance nutrient absorption, and thereby improve growth efficiency. Since *Lactobacillus* predominates in the small intestine, it is known that lactobacilli are beneficial bacteria associated with poultry health, helping to maintain intestinal barrier function and enhance nutrient absorption [51]. These bacteria also produce metabolites such as SCFAs, which are essential for maintaining intestinal integrity and stimulating immune function. Therefore, we hypothesize that the improved growth performance in broilers after MCE supplementation may be mediated through the modulation of the gut microbiota.

Alpha and beta diversity analyses revealed that MCE decreased gut microbial richness while increasing diversity and coverage, suggesting that the extract modulates microbial composition, contributing to immunity and antioxidant functions. Liu et al. [19] found that Elsholtzia ciliata (JXR) restored gut microbiota balance in DSS-treated mice, reducing *Streptococcaceae* abundance, likely due to phenolic components such as rosmarinic acid, which exhibit quorum-quenching effects. Bifidobacterium and Bacteroides levels were significantly elevated in JXR-treated mice. Moreover, dietary rosemary extract (750 mg/kg) significantly reduced Lachnoclostridium, Escherichia_Shigella, and Marvinbryantia in broilers, genera that negatively correlate with antioxidant and immune parameters, potentially enhancing broiler immunity and antioxidant capacity [44].

These findings suggest that MCE influences broiler gut microbiota by increasing SCFA production and upregulating tight junction protein expression, thereby supporting gut health and enhancing immunity and antioxidant capacity.

## 5. Conclusions

This study demonstrates that MCE improves growth performance in broilers, enhances feed intake, and promotes lipid metabolism, while also exerting antioxidant effects within the body. Serum immunological assays revealed a significant enhancement in immune factor levels with Elsholtzia ciliata supplementation. Moreover, 16S rRNA sequencing indicated that MCE alters the richness of microbial species in the small and large intestines, optimizes the gut microbiota composition, promotes short-chain fatty acid production, and increases the relative mRNA expression of tight junction proteins. In conclusion, adding MCE to a basal diet supports broiler growth and helps maintain gut health.

## Figures and Tables

**Table 1:** Vaccine immunization program.

Age	Vaccine	Immunization Method	Immunization Purpose
1 day old	Marek’s disease virus vaccine	Injection	Prevent Marek’s virus
7 days old	Newcastle disease and infectious mycoplasma combined vaccine	Eye	Prevention of Newcastle Disease Virus and Infectious Mycoplasma
14 days old	Bursal vaccine	Drinking water	Preventing Infectious Bursal Disease
20 days old	Newcastle disease and infectious mycoplasma combined vaccine	Drinking water	Prevention of Newcastle Disease Virus and Infectious Mycoplasma

**Table 2:** Feeding temperature.

Age	1 Week	2 Weeks	3 Weeks	4 Weeks	5 Weeks to Market
Temperature	33~35 °C	30~33 °C	26~29 °C	23~26 °C	21~23 °C

**Table 3:** Light duration and light intensity.

Age	1 Week	2 Weeks	3 Weeks	4 Weeks to Market
Illumination	23 h/day	22 h/day	20 h/day	18 h/day
Light intensity	20~40 lx	15~20 lx	10~15 lx	3~5 lx

**Table 4:** Humidity conditions.

Age	0~3 day	1~2 week	5 weeks
Humidity	65~70%	55~60%	55%

**Table 5:** Composition and nutritional levels of basal diet for white feather broilers.

Items	1 to 21 Days of Age	22 to 42 Days of Age
Ingredients (%)		
Corn	55.23	61.00
Soybean meal	36.00	30.00
Soybean oil	4.6	5.20
Lysine 55%	0.56	0.30
Methionine 98.5%	0.22	0.13
Dicalcium Phosphate	1.59	1.67
Limestone	1.20	1.10
Premix	0.60	0.60
Total	100.00	100.00
Nutrient levels		
Metabolizable Energy (MJ/kg)	12.76	13.18
Crude Protein (%)	21.00	19.00
Lysine (%)	1.41	1.09
Methionine (%)	0.52	0.41
Cystine (%)	1.01	0.86
Calcium	0.90	0.84
Available Phosphorus (%)	0.45	0.42

Premix for each kilogram of full-price diet: 25% copper sulfate pentahydrate: 70 mg, 30% ferrous sulfate water: 150 mg, 35% zinc sulfate monohydrate: 300 mg, 31% manganese sulfate monohydrate: 400 mg, 1% selenium: 50 mg, 1% iodine: 150 mg, multidimensional: 300 mg, choline: 500 mg, antioxidant: 100 mg, zeolite powder: 400 mg, finebran: 380 mg, phytase: 200 mg, salt: 3000 mg. Nutritional level is calculated.

**Table 6:** Fluorescent quantitative primer sequence.

Gene Name	Upstream Primer (5’ to 3’)	Downstream Primer (5’ to 3’)
β-Actin	CATCCGTAAAGACCTCTATGCCAAC	ATGGAGCCACCGATCCACA
Claudin-1	CAACGCGGGGCTGCAGCT	TTGTTTTCCGGGGACAGGA
Occludin	GGTCAGGGAATATCCACC	ATTATATTCATCAGCAGC
ZO-1	AGAAGATAGCCCTGCAGC	AGTCCGTAAGGAGATTCT

**Table 7:** Growth performance indicators.

Items	C	S1	S2	S3	SEM	*p*-Value
1–21 days ADFI/g	32.11	33.94	34.23	33.43	0.43	0.33
1–21 days ADG/g	24.10	25.00	23.95	24.80	0.29	0.52
1–21 days F/G	1.33 ^a^	1.36 ^a^	1.43 ^b^	1.35 ^a^	0.01	<0.01
22–42 days ADFI/g	106.24	118.45	108.86	116.43	2.68	0.33
22–42 days ADG/g	68.80 ^a^	75.37 ^a^	73.09^a^	80.95 ^b^	0.52	<0.01
22–42 days F/G	1.55	1.58	1.49	1.45	0.03	0.33
1–42 days ADFI/g	69.17	76.19	71.54	74.93	1.40	0.28
1–42 days ADG/g	46.45 ^a^	50.18 ^a^	48.52 ^a^	52.87 ^b^	0.97	<0.01
1–42 days F/G	1.49	1.52	1.47	1.42	0.02	0.34

Note: ADFI is the average daily feed intake; ADG is the average daily weight gain; F/G is the material to weight ratio. Different letters in the same line indicate a significant difference (*p* < 0.05).

**Table 8:** Organ index.

Items	C	S1	S2	S3	SEM	*p*-Value
Cardiac index (g/kg)	4.40	4.31	4.78	5.87	0.27	0.15
Liver index (g/kg)	17.62	16.90	18.08	19.95	0.51	0.18
Spleen index (g/kg)	1.25	1.30	1.26	1.30	0.06	0.99
Lung index (g/kg)	4.33	4.32	4.44	5.13	0.22	0.56
Renal index (g/kg)	2.92 ^a^	3.30	5.60 ^b^	4.71 ^b^	0.32	<0.01
Bursa index (g/kg)	1.41	1.59	2.13	2.27	0.18	0.06
Thymus index (g/kg)	1.41 ^a^	2.28 ^b^	1.81	2.68 ^b^	0.14	<0.01

Note: Different letters in the same line indicate a significant difference (*p* < 0.05).

**Table 9:** Serum biochemical indicators.

Items	C	S1	S2	S3	SEM	*p*-Value
UA (mmol/L)	214.67	268.17	238.67	218.33	9.21	0.15
AST (U/L)	506.10	496.61	344.16	483.33	24.76	0.06
ALT (U/L)	9.55 ^a^	6.60 ^b^	2.35 ^b^	4.68 ^b^	0.67	<0.01
ALP (U/L)	1524.50	1757.71	1763.22	1242.91	84.55	0.09
TP (g/L)	34.37	32.44	35.60	31.92	0.51	0.29
ALB (g/L)	11.46 ^a^	13.26 ^b^	14.71 ^b^	13.36 ^b^	0.28	<0.01
CK (U/L)	14,464.17	13,484.33	13,912.00	13,705.17	334.56	0.78
LDH (U/L)	2711.00 ^a^	3087.00	2206.67 ^b^	2559.00	90.01	0.03
TC (mmol/L)	3.56	3.23	3.52	3.26	0.07	0.239
TG (mmol/L)	1.82 ^a^	1.19 ^b^	1.26 ^b^	1.57	0.08	<0.01
LDL-C (mmol/L)	1.04	0.65	0.93	0.91	0.07	0.18
HDL-C (mmol/L)	2.09 ^a^	2.56 ^b^	2.83 ^b^	2.65^b^	0.08	<0.01
GLU (mmol/L)	11.37	13.00	13.38	12.96	0.32	0.12

Note: Different letters in the same line indicate a significant difference (*p* < 0.05).

## Data Availability

Data are contained within the article. NCBI number for gut microbiota: PRJNA1181119 (https://www.ncbi.nlm.nih.gov/bioproject/1181119, accessed on 20 October 2024).
